# Feasibility of Using a Flex Constant to Monitor Implant Stability Changes in a Porcine Model

**DOI:** 10.3390/s26175516

**Published:** 2026-08-31

**Authors:** Yen-Wei Chen, Weiwei Xu, Alireza Sadr, Kanako Nagatomo, Garrett Porter, Irving S. Scher, I-Chung Wang, I. Y. Shen

**Affiliations:** 1 Department of Restorative Dentistry, University of Washington, Seattle, WA 98195, USA; arsadr@uw.edu; 2 Department of Mechanical Engineering, University of Washington, Seattle, WA 98195, USA; wxu@quiver-dental.com (W.X.); ishen@uw.edu (I.Y.S.); 3 Department of Periodontics, University of Washington, Seattle, WA 98195, USA; nagakanauw@gmail.com (K.N.); icwang@uw.edu (I.-C.W.); 4 Guidance Engineering and Applied Research, Seattle, WA 98105, USA; porter@guidanceengineering.com (G.P.); scher@guidanceengineering.com (I.S.S.)

**Keywords:** dental implants, stability, flex constant, displacement–force ratio, porcine model, implant stability quotient (ISQ)

## Abstract

This experimental study investigated the feasibility of using a flex constant (displacement-to-force ratio) to monitor changes in dental implant stability in a porcine model. The stability change was incurred by thawing a porcine model from a frozen state. The changes were simultaneously monitored using resonance frequency analysis (RFA) for comparison. A Straumann BL ∅4.1×10 mm SLA implant was placed in the retromolar area of a fresh porcine mandible following the manufacturer’s surgical protocol, with the motor set at a torque of 35 N·cm. The porcine mandible was frozen to create a high-stability condition and thawed at room temperature to yield a low-stability condition. Before thawing, a regular connection (RC) locator abutment, 6 mm in length, was connected to the implant platform and secured with a torque of 10 N·cm. A custom-made motor-sensor unit, comprising a haptic unbalanced motor and a digital accelerometer, was press-fit onto the locator abutment. The motor applied a harmonic force (F) to the abutment–implant system, and the accelerometer measured the corresponding displacement (∆x). The flex constant was obtained as ∆x/F. Implant stability quotient (ISQ) was also measured using Penguin RFA for comparison. To demonstrate the feasibility, only one mandible with one implant was tested. Three rounds of tests were performed to ensure repeatability. For all three rounds of tests, ISQ dropped from high values (>80) in the frozen state to a low value (65–75) in the thawed state, occurring about 30–40 min after thawing. The drop in ISQ indicated reduction in implant stability when the frozen mandible thawed. The flex constant increased from 0.4 to 0.5 μm/N in the frozen state to 0.9–1.8 μm/N in the thawed state, with the transition occurring 30–50 min after thawing began. The increase in the flex constant implied larger displacement under the same force, indicating reduced stability. A transition peak was also observed in the flex constant measurement between the frozen and thawed states, likely due to thermal expansion and moisture condensation at the interface between the motor-sensor unit and the locator abutment. Test results from the porcine model indicate that a flex constant can reflect the changes in dental implant stability as an RFA device.

## 1. Introduction

Successful osseointegration is a prerequisite for properly functional dental implants. The state of osseointegration is often reflected in the wellness of functional and structural connections between the implant and its surrounding alveolar bone. Specifically, the wellness of the structural connection manifests as immobility of the implant, also known as implant stability [1,2]. As such, implant stability is a vital indicator to assess the well-being of an implant throughout its lifespan [3].

Currently, two major technologies are commercially available to assess implant stability. The first one is to produce a percussion between an implant abutment and a measurement device. Then characteristics of the percussion, such as contact duration and damping, are measured to infer implant stability. A representative device is Periotest, which measures the contact duration to infer implant stability. The second technology is to measure resonance frequency and convert it to an implant stability quotient (ISQ). This technology is known as resonance frequency analysis (RFA), with representative products including Osstell and Penguin RFA [3,4,5]. Although resonance frequency analysis has become one of the most widely used methods for assessing implant stability, recent reviews have suggested that the implant stability quotient (ISQ) should be regarded as a complementary clinical indicator rather than a direct biomechanical quantity of implant stability. Therefore, there remains a need for additional quantitative methods capable of providing objective physical measurements of implant stability [6,7].

Beyond contact duration and resonance frequency, researchers have long searched for alternative parameters to better quantify dental implant stability. Westover et al. [8] used linear stiffness at the implant–bone interface to represent implant stability. Pagliani et al. [9] proposed a flex constant, measured at an abutment, to indicate implant stability. Khouja et al. [10] found that angular stiffness at the implant neck accurately quantifies implant stability. Tang et al. [11] proposed the use of torsional resonance frequency as an alternative means of assessing implant stability. Emerging technologies for assessing implant stability include vibro-acoustics, electromechanical impedance [12], fractal dimension analysis [13], and damping (e.g., loss factor) [14]. Recent advances have incorporated intelligent sensing technologies, digital signal processing, and artificial intelligence into implant stability assessment to improve measurement accuracy and reliability. Nevertheless, clinically applicable methods that quantify implant stability using traceable physical quantities remain limited. Therefore, the continued development of alternative biomechanical sensing approaches is warranted [15].

Among these parameters, the flex constant is particularly intriguing for several reasons. First, it can be measured efficiently because it is the ratio of displacement to force. Physically, the flex constant is simply the reciprocal of a spring constant of the combined bone–implant–abutment system. Clinically, it can be measured using an accelerometer under harmonic force excitation applied to the implant [16]. Next, the flex constant is a physical quantity, not an artificial index like the ISQ. A subtle but profound implication of using a physical quantity is its traceability in metrology. Force and displacement measurements can be calibrated independently in benchtop tests. Therefore, the flex constant measured clinically can be calibrated and traced back to these benchtop experiments. This means that the flex constant measured in a clinical environment retains its reference because of this traceability. This is a subtle but important issue in metrology. Petersson and Sennerby [17] critiqued ISQ for lacking a defined reference quantity, noting that “implant stability per se has not been defined using any other quantity, i.e., a reference is lacking when pegs are designed and developed.” Lastly, the flex constant can serve as a precursor to other physical quantities that indicate implant stability, such as linear stiffness [8] or angular stiffness [18] at the abutment–implant junction, which are otherwise challenging to measure directly. Using a proper mathematical model, the flex constant measured at the abutment can be converted into linear or angular stiffness at the abutment–implant junction to represent implant stability [8,18]. Recent studies have also shown that angular stiffness correlates well with the insertion torque while providing a mechanical quantity expressed in physical units. Therefore, angular stiffness can potentially serve as a direct biomechanical quantity of implant stability in that ISQ may have poor correlation to resonance frequency and insertion torque [19]. Since the flex constant can be converted into angular stiffness through a mechanics-based model, these findings further support the use of flex constant measurements for the quantitative assessment of implant stability [18].

Given these potential advantages, a natural research question arises: Can a flex constant be used to monitor changes in dental implant stability in a clinical environment? Pagliani et al. demonstrated in bovine bone models that the flex constant differentiates implant mobility in bones of varying densities [9]. A natural next step is to demonstrate that the flex constant can successfully reflect a “change” of implant stability in a bone when its elastic properties change. To demonstrate such a “change”, an in vivo animal study is often required, because healing of bone around an implant is accompanied by alterations in the bone’s elastic properties [20]. In vivo animal studies, however, are expensive and take a long time to conduct. Therefore, it is very desirable to test the feasibility in a deceased animal model that the flex constant of a bone will change as the elastic property of the same bone changes. Demonstration of such feasibility will substantially increase the probability of success in future in vivo animal models.

Such feasibility study will also accelerate the development of novel sensors that measure implant stability via angular stiffness [18] or linear stiffness [8], which can be derived from a flex constant via a mechanics model. Once this feasibility is demonstrated, the probability to see a change in angular stiffness or linear stiffness in an in vivo study is substantially increased. Demonstration of this feasibility is indeed a critical step to substantially reduce the risk of future in vivo study.

This paper aims to conduct a feasibility study that the flex constant of a bone will change as the same bone’s elastic property has changed in a deceased animal model. Since the animal is deceased, its bone property does not change naturally anymore. Therefore, a major challenge in this study is to develop a repeatable experimental setup and instrumentation to induce an elastic property change in the bone of a deceased animal. This challenge is overcome by using frozen and thawed states of a porcine model to induce a change in bone elasticity. If the flex constant changes significantly between the frozen and thawed states, the feasibility is demonstrated. That means that flex constant (and thus angular stiffness) may potentially be used to monitor changes in implant stability in vivo.

To demonstrate the feasibility, a custom-made motor-sensor unit (MSU), comprising a haptic motor and a digital accelerometer, was used to measure the flex constant [18]. The haptic motor provided a known force, and the digital accelerometer recorded an acceleration. Force and acceleration data were processed to calculate the flex constant. Since bone elasticity could not be measured simultaneously and exactly during the thawing process, ISQ was measured instead (via a Penguin RFA) to indicate the change in bone elasticity for comparison. Since it was a feasibility study, only a single porcine model with a single implant was used to demonstrate the feasibility. The feasibility test was repeated three times to establish repeatability.

It is worth mentioning that the utility of MSU to measure a flex constant (and hence the angular stiffness) has been confirmed in the literature [18]. Therefore, the novelty of this study lies in the experimental setup and instrumentation developed to demonstrate the concept that the flex constant of the same bone changes as its elastic property changes in a deceased porcine model. Demonstration of the feasibility substantially reduces the risk of costly product development of a future angular stiffness sensor that may indicate implant stability for clinical applications.

## 2. Materials and Methods

### 2.1. Porcine Model Preparation

**Figure 1:**
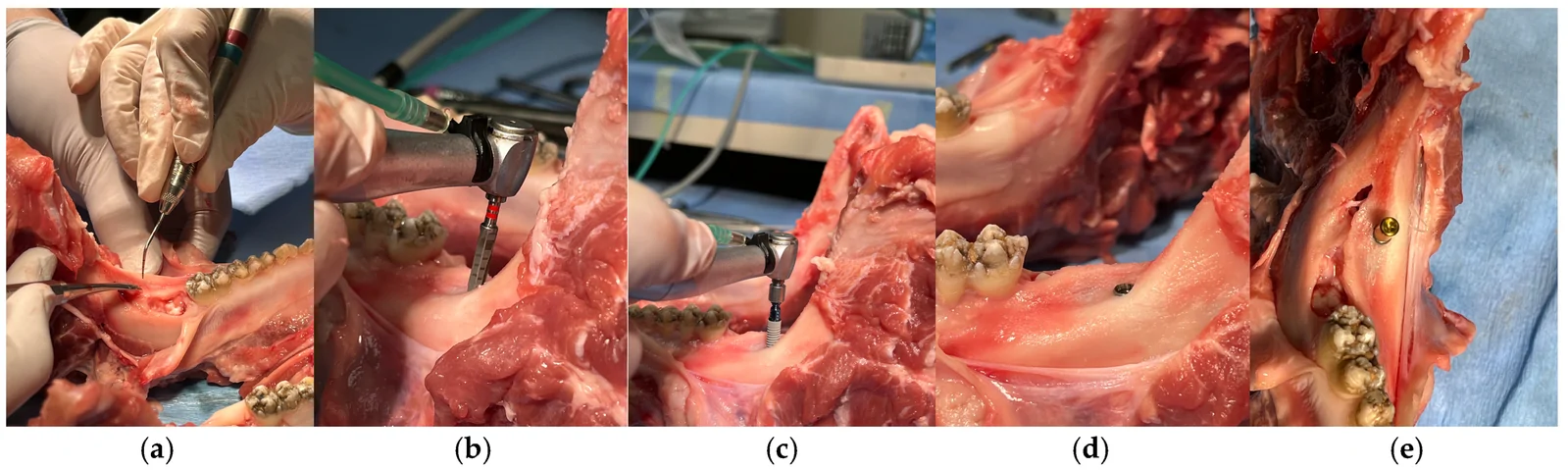
Key stages of the implant placement procedures in the porcine mandible model. (**a**) A muco-periosteal flap was reflected, and the alveolar ridge was flattened. (**b**) A sequence of drills was used to prepare the implant site following the manufacture-recommended protocol. (**c**) A Straumann BL ∅4.1×10 mm SLA implant was placed into the osteotomy site with the motor set at a torque of 35 N·cm. (**d**) The implant was placed at the level of the alveolar bone crest. (**e**) A regular connection (RC) locator abutment was connected to the implant platform.

A fresh porcine mandible was used in this in vitro implant stability study. After clinical and radiographic assessments of the anatomic structures, the implant site was determined to be in the retromolar area of the porcine mandible. A muco-periosteal flap was reflected, and the alveolar ridge was flattened (Figure 1a). A Straumann BL ∅4.1×10 mm SLA implant (Basel, Switzerland) was selected for this study. A sequence of drills was used to prepare the implant site following the manufacturer’s surgical protocol (Figure 1b). The implant was inserted into the osteotomy site with the motor set at a torque of 35 N·cm (Figure 1c) and manually placed at the level of the alveolar bone crest using the torque-controlled wrench (Figure 1d). To facilitate flex constant measurements, a regular connection (RC) locator abutment, 6 mm in length, was connected to the implant platform and secured with a torque of 10 N·cm using the dedicated wrench (Figure 1e).

### 2.2. Flex Constant Measurements

**Figure 2:**
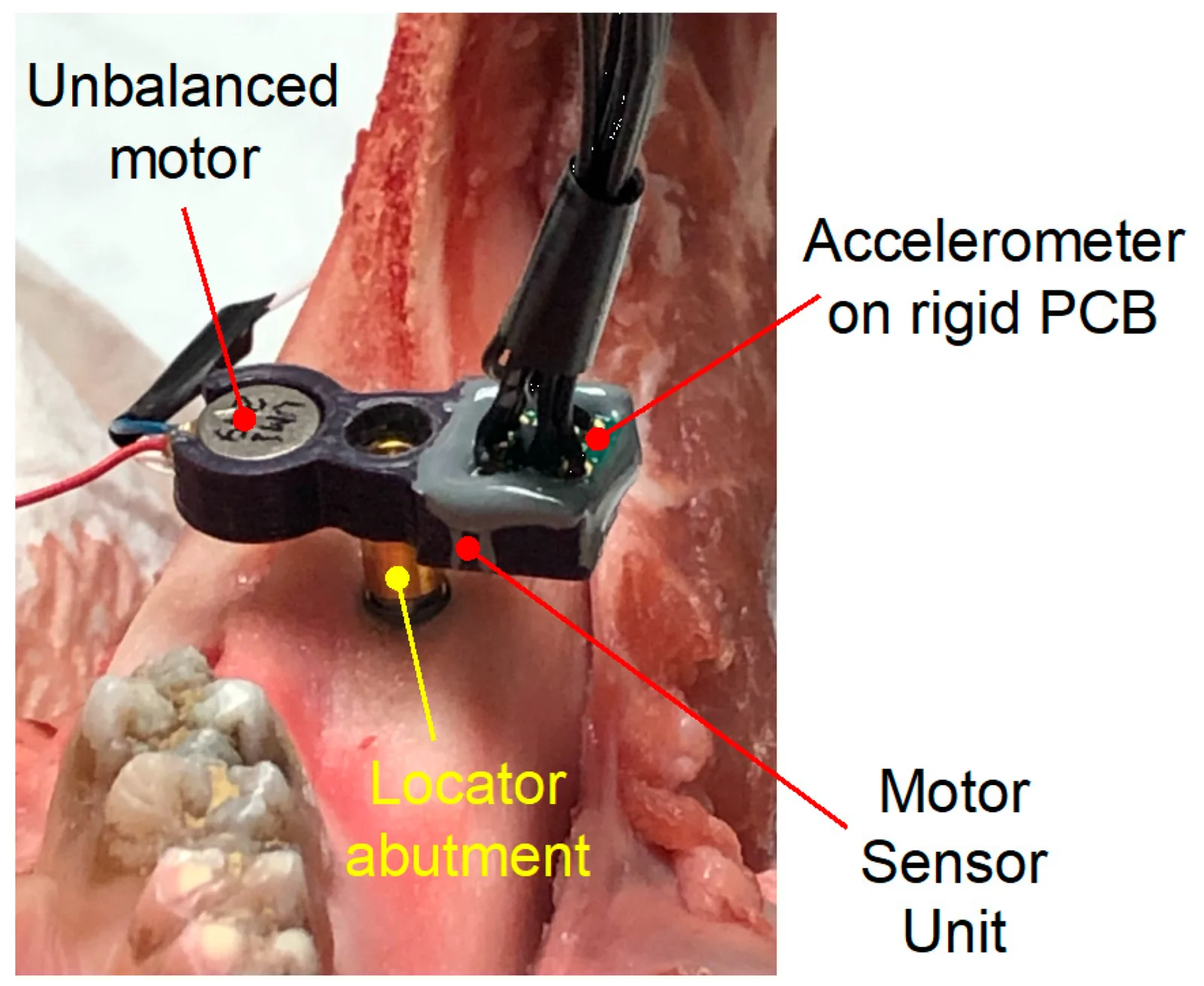
A custom-made motor-sensor unit (MSU) was press-fit onto the locator abutment to measure flex constants. The MSU comprised a brushless DC vibrator motor and an MEMS-based accelerometer. The motor generated a force, and the accelerometer measured the acceleration, from which the displacement was extracted. The ratio of the displacement to force was then used to determine the flex constant [18].

A custom-made motor-sensor unit (MSU) was press-fit onto the locator abutment to measure the flex constant (Figure 2). The MSU comprised a motor and an accelerometer. The motor was a brushless DC coin vibrator motor (Vybronics, New York, NY, USA, VW0825AB001G, ∅ 8 mm × 2.5 mm). As the motor spun at speed ω, it generated a force $F=mr\omega^{2}$, where m is the eccentric mass of the motor and r is the eccentricity. The unbalance of the motor used was $mr\;=\;8.5\;\times \;10^{-8}$ kg·m. The accelerometer was an MEMS-based triaxial accelerometer (BMA456, Bosch Sensortec GmbH, Reutlingen, Germany), surface-mounted onto a rigid printed circuit board (PCB), with an overall footprint of approximately 3.0 mm × 3.0 mm. As the eccentric motor rotated, the measured acceleration was sinusoidal with an amplitude a and a frequency ω. The flex constant ∆x/F was then obtained as $\frac{a}{mr\omega^{4}}$. The detailed design of the custom-made MSU, including its working principle, supporting electronics, and operation, was described in the paper by Xu et al. [18].

To measure the flex constant, the motor was turned on and a block of 0.3 s of accelerometer data was taken. The recorded acceleration was sinusoidal, and its peak (maximum) and bottom (minimum) values were recorded. The difference in the average peak values and the average bottom values is twice of the acceleration amplitude a. Each alternation from peak to bottom and vice versa formed a zero crossing, and every two zero crossing defined one cycle. Dividing the number of cycles by 0.3 s gave the frequency ω. Then the formula $\frac{a}{mr\omega^{4}}$ was used to calculate the flex constant.

### 2.3. Experimental Setup and Design

**Figure 3:**
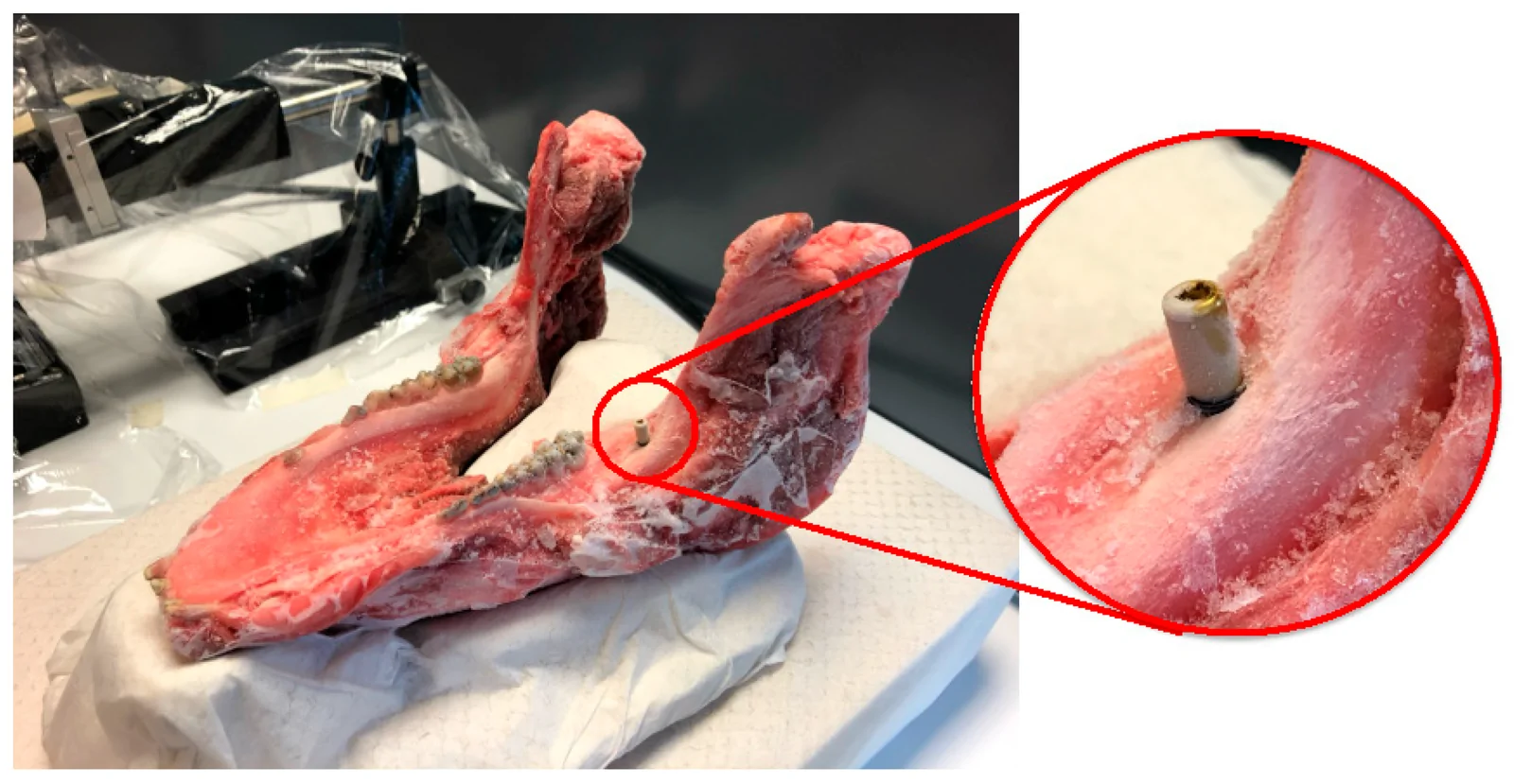
Porcine mandible in the frozen state with a blowup. The porcine mandible was placed in a chest freezer overnight to achieve a complete frozen state and then supported on a clay foundation. The locator abutment was attached onto the implant platform (cf. Figure 1e), and the custom-made MSU was press-fit onto the locator abutment (cf. Figure 2). The flex constant was measured as the mandible thawed at room temperature.

The porcine mandible, without the locator abutment, was placed in a chest freezer overnight to achieve a complete frozen state. The frozen mandible was then supported on a clay foundation (Figure 3). The locator abutment was attached to the implant platform (cf. Figure 1e), and the custom-made MSU was press-fit onto the locator abutment (cf. Figure 2). The flex constant was measured every 2–5 min as the mandible thawed at room temperature. The MSU and the locator abutment remained on the porcine mandible throughout the thawing process. The experiment was repeated in three rounds to (a) explore the best experimental setup, and (b) to ensure that the measurements and trends are consistent. Since this was a feasibility study to demonstrate the flex constant change, only one porcine mandible was used for demonstration. Statistical analysis was not performed because the purpose was to confirm if the flex constant has changed or not. It should also be noted that the three freeze–thaw rounds only demonstrated consistency of the overall trend but did not constitute repeated measurements under stable conditions. There were several reasons prohibiting the experiments from being repeated under stable conditions. First, the three rounds of freeze–thaw experiments were performed in three different days, but the room temperature was not controlled. Second, the flex constant value highly depends on where the MSU is located on the abutment. It was impossible to locate the MSU on the abutment at exactly the same location for three rounds of the test. Therefore, variations in MSU location became a noise source in the three freeze–thaw rounds.

### 2.4. ISQ Measurements

**Figure 4:**
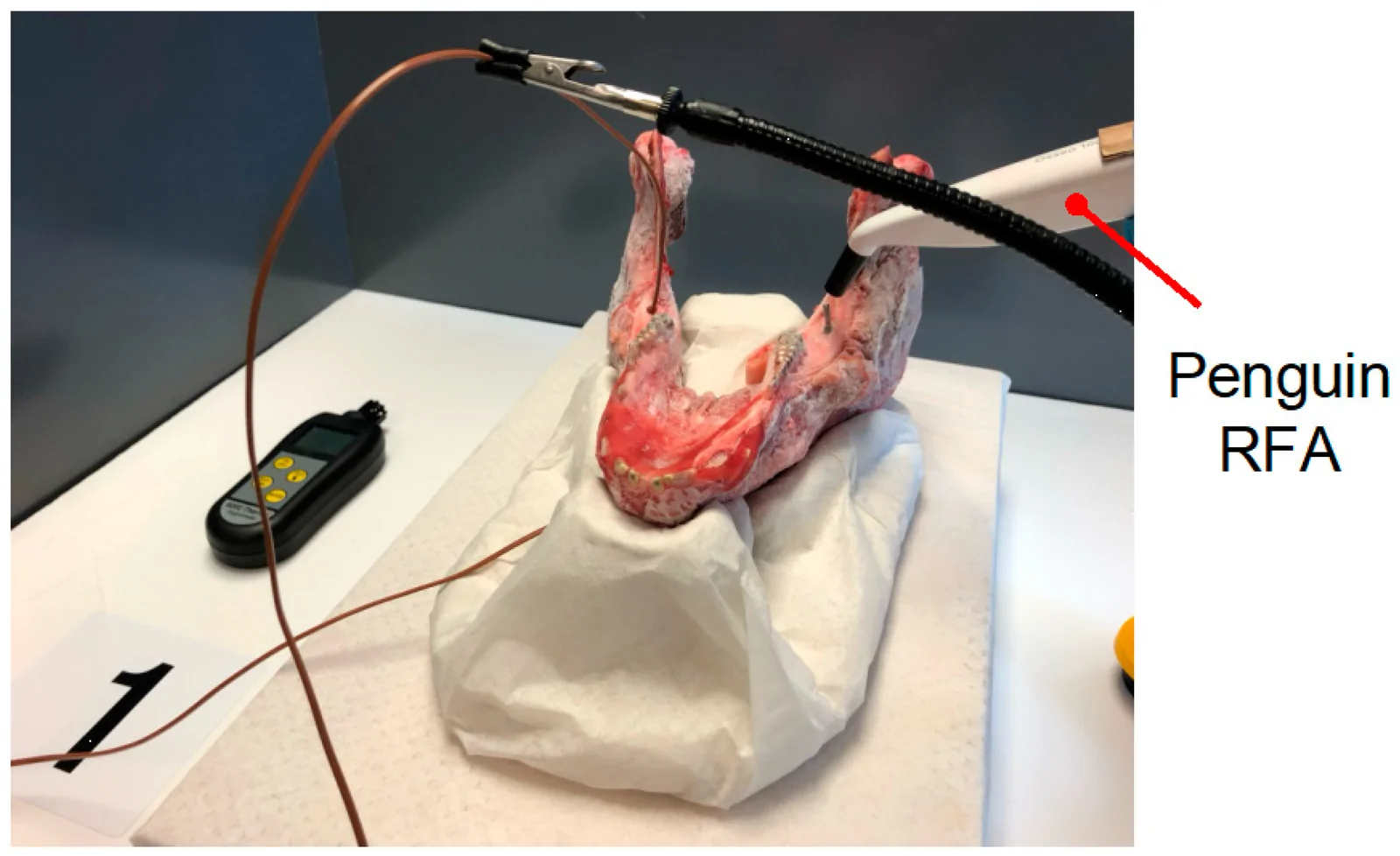
A MultiPeg was attached onto the implant in the frozen porcine mandible. A Penguin RFA was used to measure the ISQ periodically as the porcine mandible thawed at room temperature. The MultiPeg was removed after each ISQ measurement to prevent ice buildup on its surface. Two thermocouples were used to measure temperature at two reference sites.

Since the elasticity (e.g., Young’s modulus) of the porcine mandible in the frozen and thawed states cannot be measured accurately and instantaneously, an alternative way to monitor the mandible elasticity is through use of ISQ. With all other conditions remaining the same, a change in ISQ indicates a change in Young’s modulus of bone surrounding the implant and thus the implant stability. To measure ISQ, the porcine mandible, without the locator abutment, was placed in a chest freezer overnight to achieve a complete frozen state. The frozen mandible was then supported on a clay foundation (Figure 4). Before each ISQ measurement, a MultiPeg was attached onto the implant platform, and a Penguin RFA was used to measure the ISQ. The MultiPeg was removed after each measurement to prevent ice buildup. ISQ measurements were repeated every 2–5 min as the porcine mandible thawed at room temperature. The experiment was repeated in three different rounds to achieve the best experimental setup described above. Also, the three rounds of ISQ measurements would ensure that the measurements and trends were consistent.

### 2.5. Temperature Measurements

**Figure 5:**
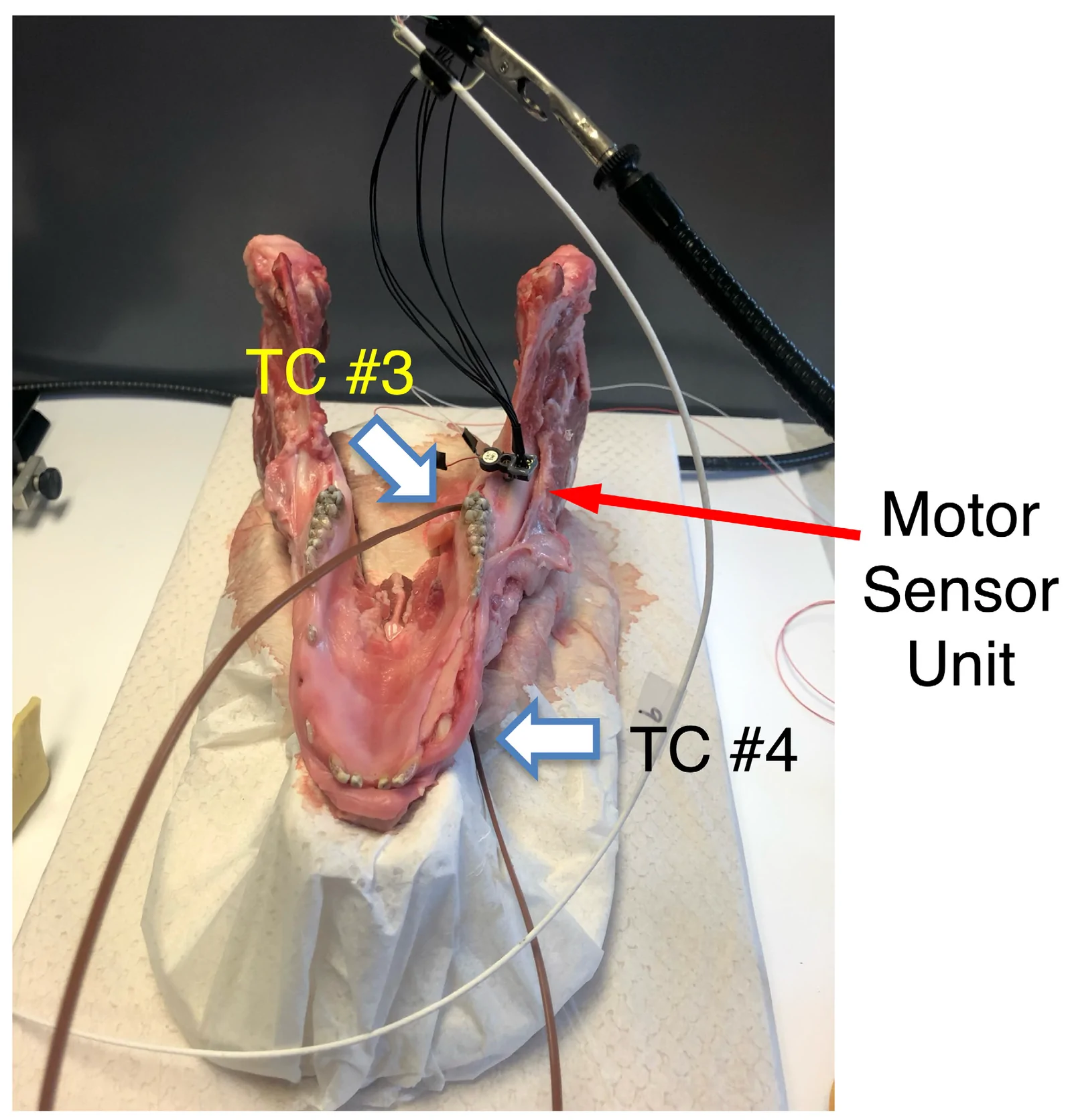
Two thermocouples were used to measure temperature at two reference sites. For flex constant measurements, the two thermocouples were placed on the same side of the implant. One was placed in a hole drilled on the mandible. The other was placed underneath the mandible.

In the original design of the experiment, mandible temperature was also measured as a monitoring reference (Figure 4 and Figure 5). For ISQ measurements, two thermocouples were placed at the opposite side of the implant (e.g., locations #1 and #2 in Figure 4) to minimize possible interference with the ISQ measurements. One was placed in a hole drilled on the mandible. The other was placed underneath the mandible. For flex constant measurements, the two thermocouples were placed on the same side of the implant; see locations #3 and #4 in Figure 5.

## 3. Results

### 3.1. Temperature Measurement Results

In the original design of the experiment, the temperature measurements were intended to serve as the independent variable. The temperature measurements, however, faced many challenges. Consistent installation and placement of thermocouples onto the mandible to obtain reliable and accurate temperature readings could not be achieved. TC#3 and #4 had very different readings, indicating a significant temperature gradient present in the mandible. Therefore, TC#3 may not properly reflect the temperature at the implant. (TC#3 could not be placed too close to the implant. Otherwise, the hole receiving TC#3 would modify the bone stiffness surrounding the implant.) Meaningful temperature measurements were only available for Round 1 of the experiment of the flex constant. The temperature measurements were abandoned. Instead, the thawing time was used as an independent variable for the rest of the feasibility study.

### 3.2. ISQ Test Results

**Figure 6:**
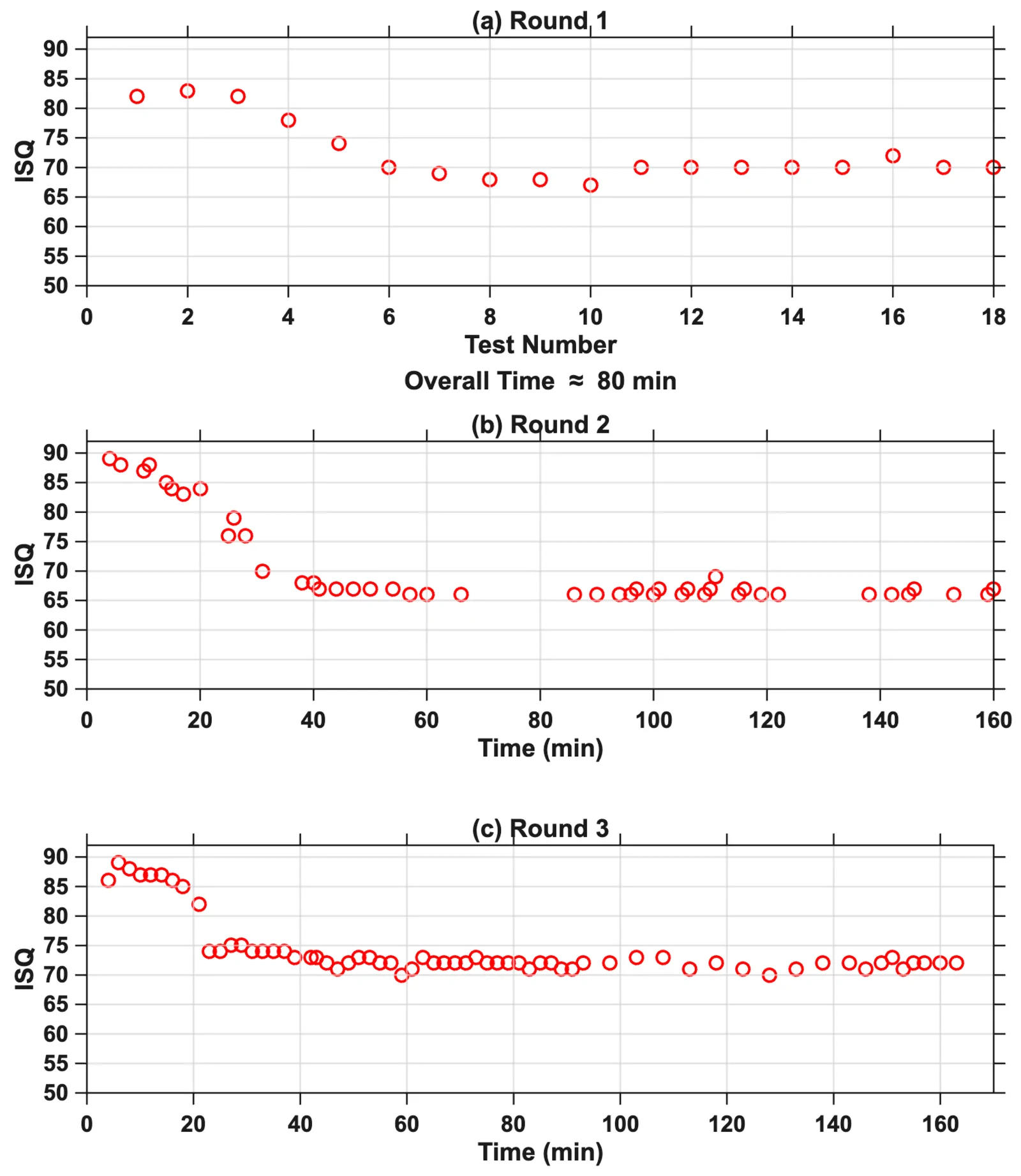
Measured ISQ values for all three rounds of experiments. (**a**) In Round 1, the test number served as the in-dependent variable. The ISQ values started at 82–83 in the frozen state and transitioned to approximately 70 in the thawed state. (**b**) In Round 2, the actual elapsed time served as the independent variable. The ISQ values started at 87–89 in the frozen state and transitioned to 66–67 in the thawed state. (**c**) In Round 3, the ISQ values started at 88–89 in the frozen state and transitioned to 71–73 in the thawed state. In all three rounds of experiments, the ISQ val-ues decreased steadily as the frozen mandible thawed, indicating a reduction in implant stability.

Figure 6a–c show measured ISQ values in the three rounds of experiment, respectively. In Round 1 of the experiment (cf. Figure 6a), the authors were exploring the best configuration to measure ISQ while struggling to obtain reliable temperature measurements. In the end, the only independent variable available was the test number, reflecting neither time nor temperature. Nevertheless, the ISQ started with 82–83 at the beginning of the experiment (i.e., the frozen state) and transitioned to roughly 70 toward the end of the experiment (i.e., the thawed state) indicating a change in bone stiffness.

In Round 2 of the experiment, temperature measurements were abandoned and time was adopted as the independent variable; see Figure 6b. The ISQ started with 87–89 at the beginning of the experiment (i.e., the frozen state) and transitioned to 66–67 toward the end of the experiment (i.e., the thawed state), confirming the change in bone stiffness from the frozen state to the thawed state. In Round 3 of the experiment, time remained the independent variable; see Figure 6c. In the frozen state, the ISQ values were approximately 88–89. As the frozen mandible thawed, the ISQ values steadily decreased indicating a reduction in implant stability. After 30 min, the ISQ values stabilized at 71–73, corresponding to the fully thawed state.

Note that the first measured ISQ value in the frozen state was 86 and followed by 89. It was unclear whether the first ISQ value represented an anomalous initial measurement due to measurement variability or a true physical variation. All three experimental rounds consistently showed that ISQ changed from high values (>80) in the frozen state to a lower value (65–75) in the thawed state, indicating a reduction in bone stiffness and, consequently, implant stability.

### 3.3. Flex Constant Test Results

**Figure 7:**
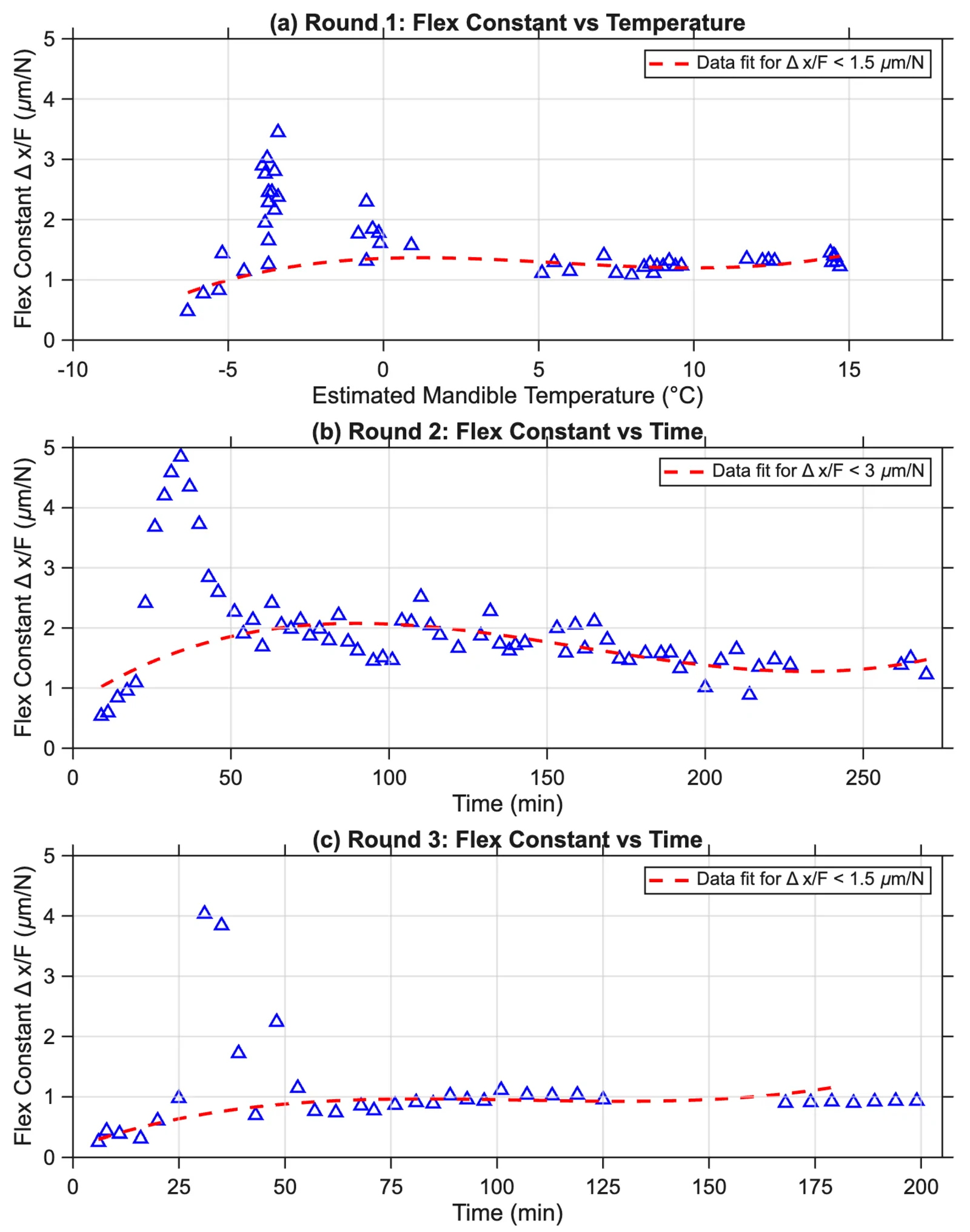
Measured flex constant as the mandible thawed for all three rounds of experiments. The flex constant response included three stages: an initial-thawing stage, a transient peak, and a fully thawed stage. (**a**) In Round 1, the measured temperature served as the independent variable. The flex constant value started at approximately 0.5 μm/N in the frozen state and transitioned to 1.2–1.5 μm/N in the thawed state. The transition peak occurred roughly between −4 and 0 °C. (**b**) In Round 2, elapsed time served as the independent variable. The flex constant value started at approximately 0.5 μm/N in the frozen state and transitioned to 1.2–1.8 μm/N in the thawed state. The transition peak occurred roughly between 30 and 45 min. (**c**) In Round 3, the flex constant value started at approximately 0.3 μm/N in the frozen state and transitioned to approximately 0.9 μm/N in the thawed state. The transition peak occurred roughly between 30 and 45 min.

Figure 7a–c shows how the measured flex constant evolved as the mandible thawed from the frozen state for all three rounds of experiments, respectively. All measured data were included and none of them were omitted or modified. In Round 1 of the experiment, the authors were able to measure the mandible temperature reliably. Therefore, the independent variable in Figure 7a is mandible temperature. For Rounds 2 and 3, the independent variable was time; see Figure 7b,c. In all three rounds of experiments, the flex constant response exhibited three stages: an initial-thawing stage, a transient peak, and a fully thawed stage. In the initial-thawing stage, the flex constant started at approximately 0.4–0.5 μm/N in the frozen state. As the mandible thawed, the flex constant gradually increased. Since the flex constant represents displacement per unit force, an increase in the flex constant indicates greater displacement and thus reduced stability.

In the fully thawed stage, the flex constant reached a steady-state range around 0.9–1.8 μm/N, depending on the round of experiments. The flex constant at the fully thawed stage was higher than that in the initial-thawing stage for every round of experiment, suggesting a clear reduction in implant stability. The stability reduction from the frozen to the thawed states in view of the flex constant measurements agreed with that revealed by the ISQ measurements. Also, the transition of ISQ and flex constant both completed roughly around 40 min after thawing began. Note that the steady-state flex constant was different for each round of test, because test conditions were not stable due to lack of room temperature control and MSU location control (cf. Section 2.3).

The transition from the initial-thawing stage to the fully thawed stage state occurred between 30 and 50 min and appeared as a sharp peak. During this period, water condensation was observed on the abutment surface. Figure 7a also indicates that the transition peak occurred when the mandible temperature was sub-zero. Once the temperature passed the freezing point, the transition peak disappeared.

## 4. Discussions

Several important issues related to the measured flex constant are discussed as follows.

### 4.1. Transient Peak of Flex Constant Measurements

The flex constant measurements were generally consistent with the ISQ measurements, except for the presence of a transient peak. The transient peak is likely attributable to the evolution of interfacial conditions between the custom-made MSU and the locator abutment. The custom-made MSU relies on a press-fit attachment to the locator abutment, creating a tight interface with sufficient frictional and normal forces. The tight interface is essential to ensure proper transmission of both force and acceleration. When the interface is somewhat weakened, the transmitted forces remain adequate, but the transmitted acceleration decreases, resulting in an apparent increase in the flex constant. Once the interfacial conditions are restored, the acceleration transmission also recovers, and the flex constant correspondingly decreases.

During the thawing processing, several mechanisms may contribute to the weakening of the interfacial conditions. One possible cause is thermal expansion. The MSU housing and the locator abutment are made of plastics and titanium, respectively. As heat transfer occurs during the thawing process, the abutment may remain at a lower temperature than the MSU housing for a short period before both reach the final steady-state temperature. In this period, the titanium locator abutment contracts more than the plastic MSU housing, thus reducing the interference fit and weakening the interfacial conditions. Another potential factor is water condensation. A tiny amount of frost condensed on the abutment may enter the interface via capillary force, thereby weakening the interface and worsening the interfacial condition. These potential contributing factors remain hypothetical and will require additional experimental investigation to confirm.

Since a tight interference is required between the custom-made MSU and the locator abutment, it is not practical to remove and attach the MSU off and on the abutment for every measurement. The tight interference prevents the MSU from staying at the same spot on the abutment every time. As a result, the MSU must stay on the abutment during the entire thawing to ensure the accuracy and consistency of the flex constant measurements. In contrast, the MultiPeg does not need to stay on the abutment continuously during the entire thawing to obtain the ISQ measurements. Other experimental variables, including ambient humidity, thawing rate, hydration state, and the mechanical properties of the clay support, were not independently quantified or controlled and may have contributed to measurement variability. These factors should be systematically investigated in future studies to further improve the control and reproducibility of the experimental setup.

### 4.2. Comparison with Published Data

One can also use prior experimental data from Pagliani et al. [9] to confirm the accuracy of the flex constant measurements in the present study. In their study, a 25 N force was applied to a 12 mm abutment in room temperature to measure the flex constant, which was then compared to ISQ measurements. When the ISQ values were 69.0±11.9, the measured displacement ranged from 48 to 85 μm, and the corresponding flex constant was 1.92–3.40 μm/N [9]. If the measurement point was at 6 mm abutment, as in the current study, the flex constant would be halved to 0.96–1.70 μm/N, which falls within the same range as the flex constant measured in the fully thawed state in the present study. Note that the scaling of the flex constant from 12 mm to 6 mm is a reasonable back-of-the-enveloped calculation. The Young’s modulus of the abutment is at least one-order-of-magnitude larger than that of the bone. Therefore, the abutment moves like a rigid stick in an elastic foundation. Most of the abutment’s motion measured results from the elastic deformation of the bone. The scaling from 12 mm to 6 mm is appropriate because the abutment barely deforms.

### 4.3. Limitation and Prospects of Flex Constants

The comparison above also shows the limitation of flex constant measurements, that is, the flex constant measurements depend on the location of the force application point and the measurement point. When those points change, the measured flex constants also change accordingly even though the implant stability itself remains the same. Therefore, the flex constant itself cannot completely define stability unless the force application point and the displacement measurement point are both fixed. This limitation suggests that a more fundamental biomechanical parameter may be needed to characterize implant stability. A better physical quantity to indicate implant stability may be linear stiffness or angular stiffness at the implant–bone interface, which does not depend on the locations of the force and displacement. The present study demonstrates that the flex constant can detect changes in implant stability and therefore provides a useful biomechanical parameter for the future development of quantitative methods for the objective assessment of implant stability.

On the other hand, measuring flex constants may offer new prospects for implant dentistry. For example, the flex constant might be useful in estimating how much an implant is moving with respect to bone under a lateral force. Estimating such micromotion of an implant in bone could be a useful method to assess the feasibility of immediate loading. Another example is the evaluation of crown stability. When a restored implant experiences some movement after use, it is often not clear if the crown becomes loose or the implant is indeed loose. In this case, measuring the flex constant at different spots of the crown may provide insights to identify the loose spot. Lastly, flex constants may become a very powerful tool to estimate the stability of full-arch restorations. The prosthesis in a full-arch restoration interacts with multiple implants through their abutments [21]. In mechanics, the most effective way to model such interaction is through use of flex constants.

## 5. Conclusions

Within the limitations of this experimental feasibility study, the following conclusions can be drawn. Both ISQ and flex constant measurements detect the artificial changes in implant stability from the frozen state to the thawed state of a porcine mandible. The measured ISQ and flex constant values were consistent with those reported in the prior literature. The feasibility demonstration relies on a freeze–thaw mandible model that artificially alters elastic properties of bone; therefore, the current study merely serves as a first mechanical test before further biological validation can be performed.

## Data Availability

The data presented in this study are available from the corresponding author upon request.

## Abbreviations

ISQ, Implant Stability Quotient; MEMS, Micro-Electro-Mechanical Systems; MSU, Motor-Sensor Unit; PCB, Printed Circuit Board; RFA, Resonance Frequency Analysis.
